# Sexual function in Britain: findings from the third National Survey of Sexual Attitudes and Lifestyles (Natsal-3)

**DOI:** 10.1016/S0140-6736(13)62366-1

**Published:** 2013-11-30

**Authors:** Kirstin R Mitchell, Catherine H Mercer, George B Ploubidis, Kyle G Jones, Jessica Datta, Nigel Field, Andrew J Copas, Clare Tanton, Bob Erens, Pam Sonnenberg, Soazig Clifton, Wendy Macdowall, Andrew Phelps, Anne M Johnson, Kaye Wellings

**Affiliations:** a Centre for Sexual and Reproductive Health Research, Department of Social and Environmental Health Research, London School of Hygiene & Tropical Medicine, London, UK; b Department of Population Health, London School of Hygiene & Tropical Medicine, London, UK; c Department of Health Services Research and Policy, London School of Hygiene & Tropical Medicine, London, UK; d Research Department of Infection and Population Health, University College London, London, UK; e NatCen Social Research, London, UK

## Abstract

**Background:**

Despite its importance to sexual health and wellbeing, sexual function is given little attention in sexual health policy. Population-based studies are needed to understand sexual function across the life course.

**Methods:**

We undertook a probability sample survey (the third National Survey of Sexual Attitudes and Lifestyles [Natsal-3]) of 15 162 individuals aged 16–74 years who lived in Britain (England, Scotland, and Wales). Interviews were done between Sept 6, 2010, and Aug 31, 2012. We assessed the distribution of sexual function by use of a novel validated measure (the Natsal-SF), which assessed problems with individual sexual response, sexual function in a relationship context, and self-appraisal of sex life (17 items; 16 items per gender). We assess factors associated with low sexual function (defined as the lowest quintile of distribution of Natsal-SF scores) and the distribution of components of the measure. Participants reporting one or more sexual partner in the past year were given a score on the Natsal-SF (11 690 participants). 4122 of these participants were not in a relationship for all of the past year and we employed the full information maximum likelihood method to handle missing data on four relationship items.

**Findings:**

We obtained data for 4913 men and 6777 women for the Natsal-SF. For men and women, low sexual function was associated with increased age, and, after age-adjustment, with depression (adjusted odds ratio 3·70 [95% CI 2·90–4·72] for men and 4·11 [3·36–5·04] for women) and self-reported poor health status (2·63 [1·73–3·98] and 2·41 [1·72–3·39]). Low sexual function was also associated with experiencing the end of a relationship (1·52 [1·18–1·95] and 1·77 [1·44–2·17]), inability to talk easily about sex with a partner (2·36 [1·94–2·88] and 2·82 [2·28–3·48]), and not being happy in the relationship (2·89 [2·32–3·61] and 4·10 [3·39–4·97]). Associations were also noted with engaging in fewer than four sex acts in the past 4 weeks (3·13 [2·58–3·79] and 3·38 [2·80–4·09]), having had same sex partners (2·28 [1·56–3·35] and 1·60 [1·16–2·20]), paying for sex (in men only; 2·62 [1·46–4·71]), and higher numbers of lifetime sexual partners (in women only; 2·12 [1·68–2·67] for ten or more partners). Low sexual function was also associated with negative sexual health outcomes such as experience of non-volitional sex (1·98 [1·14–3·43] and 2·18 [1·79–2·66]) and STI diagnosis (1·50 [1·06–2·11] and 1·83 [1·35–2·47]). Among individuals reporting sex in the past year, problems with sexual response were common (41·6% of men and 51·2% of women reported one or more problem) but self-reported distress about sex lives was much less common (9·9% and 10·9%). For individuals in a sexual relationship for the past year, 23·4% of men and 27·4% of women reported an imbalance in level of interest in sex between partners, and 18·0% of men and 17·1% of women said that their partner had had sexual difficulties. Most participants who did not have sex in the past year were not dissatisfied, distressed, or avoiding sex because of sexual difficulties.

**Interpretation:**

Wide variability exists in the distribution of sexual function scores. Low sexual function is associated with negative sexual health outcomes, supporting calls for a greater emphasis on sexual function in sexual health policy and interventions.

**Funding:**

Grants from the UK Medical Research Council and the Wellcome Trust, with support from the Economic and Social Research Council and the Department of Health.

## Introduction

Sexual function is an important component of quality of life. It is associated with mental and physical wellbeing and with relationship satisfaction.^1^, ^2^, ^3^, ^4^ However, sexual function is rarely explored in a public health context.^5^ It has been given little attention as a component of sexual health policy, and its association with other sexual health indicators has seldom been measured.

In recent years, research in this specialty has reported on problems with sexual function in a clinical context and has focused on physiological aspects of sexual response and clinical diagnoses of dysfunction.^6^, ^7^ This trend has intensified with advances in pharmacological treatment options, and has led to criticism of overmedicalisation of sexual experiences.^8^, ^9^

A focus on clinical pathology might neglect other important aspects of function such as the sexual relationship, the level of satisfaction, and the significance of problems for the individual concerned.^10^ At a population level, surveys tend to measure sexual problems separately from these other aspects of function. Population surveys either use single items^11^ or clinical measures, partly because no brief measure exists (with male and female versions) specifically designed for community surveys.^12^ Many measures are designed as endpoints in clinical trials^13^ and focus on biomedical aspects of dysfunction; and few have involved patients in their development.

In this study we explore the distribution of, and factors associated with, sexual function in a general population sample. We use a psychometrically validated measure of sexual function, developed for use in the third National Survey of Sexual Attitudes and Lifestyles (Natsal-3),^14^ which is applicable to both men and women, and which takes account of the variability of sexual response according to the relationship context and the subjective significance for the individual concerned. This measure, the Natsal-SF, is derived from qualitative interviews with individuals in the community and patients in a sexual problems clinic.^15^, ^16^ In this qualitative work, we conceptualised sexual function as the extent to which an individual is able to participate in and enjoy a sexual relationship.

## Methods

### Participants and procedures

Natsal-3 was a stratified probability sample survey of 15 162 men and women aged 16–74 years in Britain (England, Scotland, and Wales), interviewed between Sept 6, 2010, and Aug 31, 2012. The estimated response rate was 57·7%, while the cooperation rate was estimated at 65·8% (of all eligible addresses contacted). Participants completed the survey through a combination of computer-assisted face-to-face interviews and self-interview. Details of the survey methods and response calculations are described elsewhere.^17^, ^18^, ^19^ An anonymised dataset will be deposited with the UK Data Archive, and the complete questionnaire and technical report will be available on the Natsal website on the day of publication.

The Natsal-3 study was approved by the Oxfordshire Research Ethics Committee A (10/H0604/27). Participants provided oral informed consent for interviews.

We assessed sexual function in Britain with the Natsal-SF,^14^ a newly developed measure of sexual function comprising components on problems with sexual response, sexual function in the relationship context, and self-appraisal of sex life (panel 1).

Panel 1 **Components of the Natsal-SF**

Component one: problems with sexual response (participants were asked to report which, if any, of the following sexual difficulties they had had for a period of 3 months or more in the past year)

• Lacked interest in having sex
• Lacked enjoyment in sex
• Felt anxious during sex
• Felt physical pain as a result of sex
• Felt no excitement or arousal during sex
• Did not reach a climax (experience an orgasm) or took a long time to reach a climax despite feeling excited or aroused
• Reached climax (experienced an orgasm) more quickly than you would like
• Had an uncomfortably dry vagina (asked of women only)
• Had trouble getting or keeping an erection (asked of men only)

Component two: sexual function in relationship context (participants were asked to think about their sexual relationship in the past year)

• My partner and I share about the same level of interest in having sex (response options: agree strongly, agree, neither agree nor disagree, disagree, disagree strongly)
• My partner and I share the same sexual likes and dislikes (response options: agree strongly, agree, neither agree nor disagree, disagree, disagree strongly)
• My partner has had sexual difficulties in the past year (response options: agree strongly, agree, neither agree nor disagree, disagree, disagree strongly)
• How often would you say you feel emotionally close to your partner when you have sex together? (options: always, most of the time, sometimes, not very often, hardly ever)

Component three: appraisal of sex life (participants were asked to think about their sex life in the past year)

• I feel satisfied with my sex life (response options: agree strongly, agree, neither agree nor disagree, disagree, disagree strongly)
• I feel distressed or worried about my sex life. (response options: agree strongly, agree, neither agree nor disagree, disagree, disagree strongly)
• I have avoided sex because of sexual difficulties, either my own or those of my partner (response options: agree strongly, agree, neither agree nor disagree, disagree, disagree strongly)
• Have you sought help or advice regarding your sex life from any of the following sources in the last year? (participants selected all that applied from a list of ten sources; four informal sources [including family member or friend, information and support sites on internet] and six professional sources [including GP or family doctor, sexual health clinic, genitourinary clinic, sexually transmitted infection clinic, or relationship counsellor])

The 17 item measure (16 items per gender) was validated in a general population sample (an internet panel survey of 1262 participants) and a clinical sample (100 patients attending sexual problems clinics). The Natsal-SF has good discriminant validity (odds ratio [OR] 2·667 for clinical group), acceptable test-retest reliability (*r*=0·72), and good model fit, both in the validation study^14^ and in the Natsal sample (comparative fit index 0·967, values >0·95 signify very good fit; Tucker Lewis index 0·965, values >0·95 signify very good fit; and root mean square error of approximation 0·037, values <0·06 signify very good fit).

**Figure 1:**
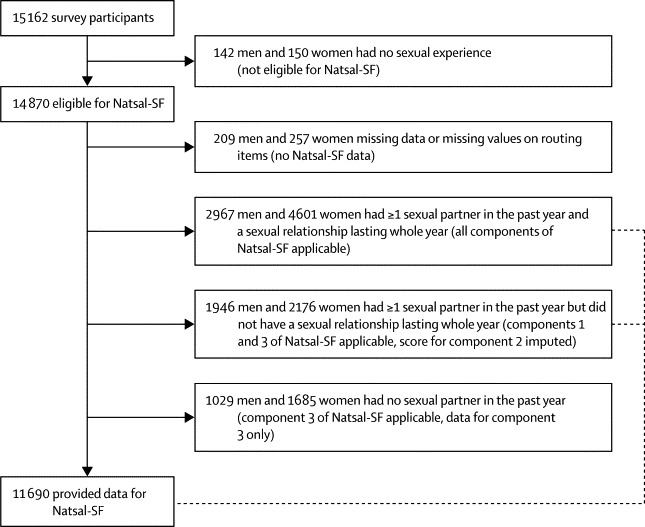
Natsal-SF participants

Routing of participants to and within the Natsal-SF depended on their sexual activity and relationship status (figure 1). All participants who were sexually active in the past year (ie, reported one or more sexual partners in this timeframe) were given a score on the Natsal-SF derived from their responses to the items in the measure (panel 1). Individuals who were sexually active but not in a relationship for all of the past year were ineligible for component two, and we employed the full information maximum likelihood method to handle their missing data.^20^ Thus, these participants were regarded as having hypothetical relationships. Their answers concerning function in relation to their hypothetical partner were regarded to be the same as participants with partners who gave the same responses to other items in the Natsal-SF. This assumption was deemed acceptable, in part because component two comprises only four of the 17 items.^14^ We estimated factor scores for the Natsal-SF on the basis of the general specific measurement model. Each participant received a numerical score, which indicates their relative standing on the Natsal-SF. The scores are a function of the estimated model parameters and the pattern of participants' responses on the 17 items.

The distribution of scores on the Natsal-SF had no empirical threshold and no gold standard approach exists to identify low sexual function in population samples. For the purpose of testing associations, we imposed a categorical measurement on the continuum,^21^ treating the lowest quintile of the sex-specific population distribution of scores as low sexual function (ie, low function relative to the rest of the sample).

Sex was defined as vaginal, oral, or anal intercourse with an opposite-sex or same-sex partner or partners, and sex life was defined as sexual thoughts, sexual feelings, sexual activity, and sexual relationships.

### Statistical analysis

We describe the distribution of scores on the Natsal-SF in the survey sample, and then explore the association between low sexual function and selected demographic, behavioural, and sexual health variables. We also explore the prevalence of items within the Natsal-SF, by sex and age-group. We weighted Natsal-3 data before analysis to adjust for unequal probabilities of selection as described elsewhere.^19^

We undertook all analyses with the complex survey functions of STATA, version 12.1 to incorporate the weighting, clustering, and stratification of the data. To examine the association between low sexual function and a range of independent variables, we used binary logistic regression to calculate ORs adjusted for age (aAOR).

We present descriptive statistics of items within the Natsal-SF and test for significant age and sex differences with χ^2^. We use strength of association rather than a strict cutoff for statistical significance (such as p<0·05) to assess the importance of relationships in the logistic regression analyses.

### Role of the funding source

The sponsors of the study had no role in study design, data collection, data analysis, data interpretation, or writing of the report. The corresponding author had full access to all the data in the study and had final responsibility for the decision to submit for publication.

## Results

**Figure 2:**
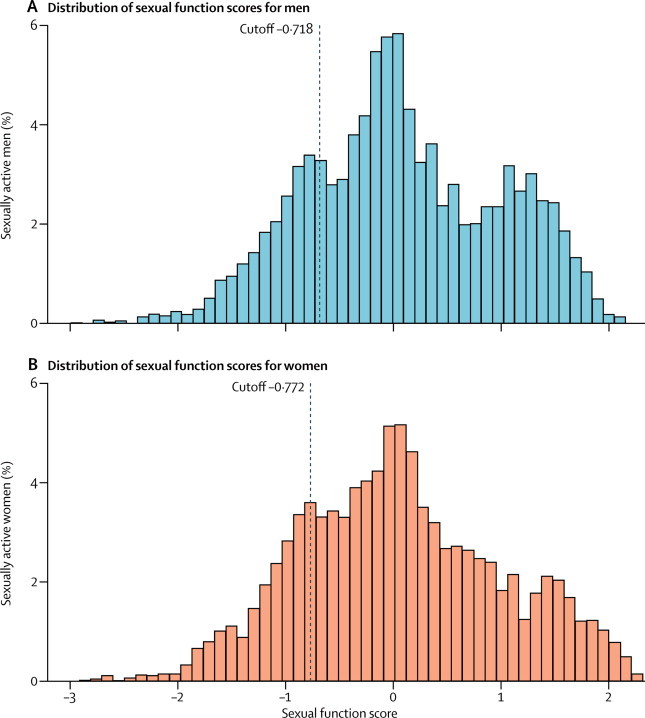
Distribution of raw latent scores according to the Natsal-SF in sexually active men and women

Figure 2 shows the distribution of Natsal-SF scores for the 4913 men and 6777 women who were sexually active in the past year and completed the Natsal-SF. Scores ranged from −2·992 to 2·163 in men and −2·914 to 2·325 in women. The black dashed line marks the lowest quintile, used as the cutoff to denote low sexual function.

We explored associations between low sexual function and sociodemographic, health, relationship, and sexual behaviour variables (Table 1, Table 2). The percentage with low sexual function increased with age in sexually active men (table 1) and women (table 2). However, the strength of the association did not increase beyond the 55–64-year-old age group.

**Table 1:** Factors associated with low sexual function (lowest quintile of gender-specific distribution) in sexually active men

		Percentage with low sexual function (95% CI)*	**Age-adjusted odds ratio (95% CI)**	**p value**	**Denominator**	
					Unweighted	Weighted
**Sociodemographic**						
Age group (years)				<0·0001		
	16–24	14·1% (12·1–16·4)	1·00	..	1291	944
	25–34	16·6% (14·5–19·0)	1·21 (0·95–1·55)	..	1380	1242
	35–44	21·2% (18·1–24·6)	1·63 (1·25–2·14)	..	721	1302
	45–54	18·4% (15·4–21·9)	1·37 (1·04–1·81)	..	639	1204
	55–64	27·8% (23·9–32·1)	2·35 (1·79–3·07)	..	515	851
	65–74	27·0% (22·5–32·1)	2·25 (1·67–3·04)	..	326	471
Quintile of Index of Multiple Deprivation†				0·5020		
	1 (least deprived)	20·6% (17·8–23·8)	1·00	..	982	1281
	2	18·6% (15·9–21·6)	0·89 (0·68–1·16)	..	968	1270
	3	21·4% (18·5–24·5)	1·10 (0·85–1·42)	..	944	1176
	4	18·9% (16·1–22·1)	0·99 (0·75–1·29)	..	977	1196
	5 (most deprived)	20·4% (17·6–23·5)	1·10 (0·85–1·42)	..	1001	1091
Employment status at interview				0·0011		
	Employed	19·0% (17·4–20·6)	1·00	..	3221	4269
	In full-time education	13·2% (10·1–17·2)	0·97 (0·68–1·38)	..	552	438
	Unemployed	26·0% (22·3–30·1)	1·55 (1·23–1·96)	..	717	739
	Retired	24·6% (20·4–29·3)	0·87 (0·64–1·18)	..	378	566
**Health**						
Current depression (PHQ-2)‡				<0·0001		
	No	17·9% (16·6–19·2)	1·00	..	4409	5500
	Yes	43·0% (37·7–48·5)	3·70 (2·90–4·72)	..	455	501
Self-reported health status				<0·0001		
	Very good or good	17·6% (16·2–19·0)	1·00	..	4149	5088
	Fair	31·7% (27·7–36·0)	2·01 (1·60–2·52)	..	582	748
	Bad or very bad	39·1% (30·0–48·9)	2·63 (1·73–3·98)	..	140	176
**Relationship context**						
Relationship status at interview				<0·0001		
	Living with a partner	20·4% (18·7–22·1)	1·00	..	2722	4289
	Steady relationship, not cohabiting	13·5% (11·4–16·1)	0·80 (0·63–1·02)	..	953	763
	No steady relationship, previously cohabited	26·2% (22·1–30·9)	1·52 (1·18–1·95)	..	451	393
	No steady relationship, never cohabited	21·4% (18·3–24·9)	1·66 (1·27–2·18)	..	736	561
Always find it easy to talk about sex with partners§				<0·0001		
	Yes	11·3% (9·7–13·2)	1·00	..	1705	1912
	No/other	24·1% (22·4–25·9)	2·36 (1·94–2·88)	..	3143	4072
Happy with relationship¶				<0·0001		
	Yes	13·0% (11·4–14·9)	1·00	..	1952	2794
	Other	30·3% (27·1–33·8)	2·89 (2·32–3·61)	..	993	1425
**Sexual behaviour and indicators of sexual health**						
Sexual competence at first intercourse‖				0·0018		
	Competent	17·0% (15·2–18·9)	1·00	..	2306	2791
	Not competent	22·4% (20·5–24·4)	1·33 (1·11–1·58)	..	2427	3063
Four or more sexual acts, past 4 weeks**				<0·0001		
	Yes	9·8% (8·5–11·4)	1·00	..	2039	2464
	No	26·5% (24·6–28·5)	3·13 (2·58–3·79)	..	2652	3338
Masturbation, past 4 weeks				<0·0001		
	No	17·5% (15·3–19·8)	1·00	..	1309	1846
	Yes	21·0% (19·4–22·6)	1·52 (1·25–1·85)	..	3550	4152
Genital contact without intercourse, past 4 weeks††				<0·0001		
	Yes	16·1% (14·4–17·9)	1·00	..	2608	3134
	No	24·1% (22·2–26·2)	1·55 (1·31–1·85)	..	2259	2872
At least one same sex partner, past 5 years				<0·0001		
	No	19·5% (18·2–20·9)	1·00	..	4699	5838
	Yes	34·4% (26·6–43·2)	2·28 (1·56–3·35)	..	173	176
Paid for sex, past year				0·0013		
	No	19·7% (18·4–21·1)	1·00	..	4805	5935
	Yes	39·4% (26·9–53·5)	2·62 (1·46–4·71)	..	66	78
Number of sexual partners, lifetime‡‡				0·0971		
	1	16·3% (13·2–20·0)	1·00	..	617	777
	2	23·7% (18·9–29·4)	1·64 (1·13–2·38)	..	384	471
	3–4	18·6% (15·5–22·1)	1·19 (0·86–1·65)	..	714	891
	5–9	20·7% (18·2–23·5)	1·35 (1·00–1·81)	..	1225	1537
	≥10	20·5% (18·5–22·8)	1·35 (1·02–1·80)	..	1888	2289
Ever had non-volitional sex§§				0·0158		
	No	19·6% (18·3–20·9)	1·00	..	4736	5862
	Yes	31·7% (21·1–44·5)	1·98 (1·14–3·43)	..	71	82
Diagnosed with an STI in the past 5 years¶¶				0·0206		
	No	19·8% (18·4–21·2)	1·00	..	4556	5703
	Yes	23·1% (17·9–29·2)	1·50 (1·06–2·11)	..	266	248

Sexually active participants are regarded as individuals who reported at least one sexual partner (opposite-sex or same-sex) in the past year. PHQ-2=Patient Health Questionnaire-2. STI=sexually transmitted infection.

* Variations from the figure of 20% (cut-off used for low sexual function) show increased or decreased sexual function with variable groups.

† A multidimensional measure of area (neighbourhood)-level deprivation based on the participant's postcode; Index of Multiple Deprivation scores for England, Scotland, and Wales were adjusted before assignment to quintiles by use of a method by Payne and Abel;^22^ this approach allowed use of single Index of Multiple Deprivation measure for the three countries.

‡ Two screening questions (scored 0–3 per question; defined here by a total score of 3 or more^24^) assessed depressive symptoms (PHQ-2),^23^ participants were asked whether they had been bothered by feeling down, depressed, or hopeless, and whether they had been often bothered by little interest or pleasure in doing things, in the previous 2 weeks.

§ Other means easy with a husband or wife or regular partner, but difficult with a new partner; easy with a new partner, but difficult with a husband or wife or regular partner; difficult with any partner, it depends, sometimes easy, and sometimes difficult.

¶ Participants were asked to rate how happy they were in their relationship from 1 (very happy) to 7 (very unhappy); responses of 1 or 2 were regarded as denoting participants who were happy with their relationship.

‖ First intercourse classified as competent if there was absence of duress and regret about timing; if there was autonomy of decision; and if a reliable form of contraception was used.^25^

** Defined as vaginal, oral, or anal intercourse.

†† Defined as genital contact not involving vaginal, oral, or anal intercourse, but intended to achieve orgasm, for example stimulating by hand.

‡‡ Female or male sexual partners, or both.

§§ Defined as anyone having sex with you against your will after the age of 13 years.

¶¶ Diagnosed with chlamydia, gonorrhoea, herpes, genital warts, trichomonas, non-specific or non-gonococcal urethritis, or syphilis.

**Table 2:** Factors associated with low sexual function (lowest quintile of gender-specific distribution) in sexually active women

		Percentage with low sexual function (95% CI)*	**Age-adjusted odds ratio (95% CI)**	**p value**	**Denominator**	
					Unweighted	Weighted
**Sociodemographic**						
Age group (years)				<0·0001		
	16–24	13·2% (11·4–15·1)	1·00	..	1677	931
	25–34	16·4% (14·7–18·3)	1·30 (1·06–1·59)	..	2243	1250
	35–44	20·4% (17·9–23·1)	1·68 (1·35–2·10)	..	1054	1298
	45–54	22·9% (20·0–26·1)	1·96 (1·56–2·46)	..	877	1197
	55–64	27·3% (23·4–31·4)	2·47 (1·91–3·19)	..	574	761
	65–74	24·1% (19·0–30·1)	2·10 (1·49–2·95)	..	286	362
Quintile of Index of Multiple Deprivation†				0·4772		
	1 (least deprived)	21·3% (18·6–24·2)	1·00	..	1254	1214
	2	18·6% (16·1–21·3)	0·86 (0·68–1·09)	..	1297	1212
	3	19·7% (17·1–22·7)	0·98 (0·76–1·25)	..	1305	1122
	4	20·4% (18·0–23·0)	1·04 (0·83–1·31)	..	1400	1157
	5 (most deprived)	20·2% (17·6–22·9)	1·05 (0·82–1·33)	..	1455	1094
Employment status at interview				0·1119		
	Employed	19·2% (17·7–20·8)	1·00	..	3889	3540
	In full-time education	16·0% (13·0–19·7)	1·21 (0·91–1·61)	..	702	428
	Unemployed	21·1% (18·9–23·5)	1·19 (1·01–1·41)	..	1692	1294
	Retired	25·8% (21·5–30·7)	0·86 (0·64–1·16)	..	419	529
**Health**						
Current depression (PHQ-2)‡				<0·0001		
	No	17·2% (16·0–18·5)	1·00	..	5917	5181
	Yes	43·9% (39·7–48·3)	4·11 (3·36–5·04)	..	788	612
Self-reported health status				<0·0001		
	Very good or good	17·9% (16·6–19·2)	1·00	..	5717	4885
	Fair	30·1% (26·4–34·1)	1·83 (1·49–2·24)	..	787	717
	Bad or very bad	37·0% (29·8–44·9)	2·41 (1·72–3·39)	..	207	197
Menopausal status§				<0·0001		
	Not menopausal	17·9% (16·7–19·2)	1·00	..	5516	4215
	Menopausal	25·6% (23·0–28·5)	1·58 (1·34–1·86)	..	1195	1584
Pregnant in the past year				0·0074		
	No	20·7% (19·5–22·1)	1·00	..	5824	5238
	Yes	13·1% (10·7–15·9)	0·72 (0·57–0·92)	..	868	544
**Relationship context**						
Relationship status at interview				<0·0001		
	Living with a partner	20·6% (19·1–22·1)	1·00	..	3994	4203
	Steady relationship, not cohabiting	12·0% (10·1–14·2)	0·66 (0·53–0·82)	..	1364	790
	No steady relationship, previously cohabited	29·6% (25·9–33·6)	1·77 (1·44–2·17)	..	756	467
	No steady relationship, never cohabited	19·1% (15·7–23·0)	1·36 (1·04–1·78)	..	587	334
Always find it easy to talk about sex with partners¶				<0·0001		
	Yes	9·7% (8·1–11·5)	1·00	..	1759	1463
	No/other	23·5% (22·1–25·1)	2·82 (2·28–3·48)	..	4933	4318
Happy with relationship‖				<0·0001		
	Yes	10·7% (9·3–12·2)	1·00	..	2738	2604
	Other	33·3% (30·5–36·1)	4·10 (3·39–4·97)	..	1638	1615
**Sexual behaviour and indicators of sexual health**						
Sexual competence at first intercourse**				<0·0001		
	Competent	15·5% (14·0–17·2)	1·00	..	3111	2731
	Not competent	24·2% (22·5–26·0)	1·71 (1·47–1·98)	..	3459	2952
Four or more sexual acts, past 4 weeks††				<0·0001		
	Yes	9·2% (7·9–10·7)	1·00	..	2664	2203
	No	26·9% (25·1–28·7)	3·38 (2·80–4·09)	..	3585	3243
Masturbation, past 4 weeks				0·0030		
	No	19·2% (17·8–20·7)	1·00	..	4061	3642
	Yes	21·4% (19·4–23·5)	1·26 (1·08–1·46)	..	2621	2121
Genital contact without intercourse, past 4 weeks‡‡				<0·0001		
	Yes	15·0% (13·5–16·5)	1·00	..	3523	2894
	No	25·1% (23·3–27·0)	1·76 (1·51–2·04)	..	3164	2878
At least one same sex partner, past 5 years				0·0039		
	No	19·8% (18·6–21·1)	1·00	..	6386	5576
	Yes	25·2% (19·9–31·5)	1·60 (1·16–2·20)	..	325	224
Number of sexual partners, lifetime§§				<0·0001		
	1	15·5% (13·2–18·2)	1·00	..	1214	1208
	2	19·1% (15·9–22·7)	1·34 (1·00–1·79)	..	688	627
	3–4	19·2% (16·6–22·0)	1·39 (1·08–1·78)	..	1244	1116
	5–9	20·6% (18·4–23·1)	1·60 (1·27–2·01)	..	1778	1484
	≥10	24·6% (22·2–27·2)	2·12 (1·68–2·67)	..	1741	1322
Ever had non-volitional sex¶¶				<0·0001		
	No	18·4% (17·2–19·7)	1·00	..	5857	5097
	Yes	32·7% (28·8–36·9)	2·18 (1·79–2·66)	..	683	579
Diagnosed with an STI in the past 5 years‖‖				0·0001		
	No	19·7% (18·5–21·0)	1·00	..	6270	5534
	Yes	24·5% (19·5–30·2)	1·83 (1·35–2·47)	..	398	229

Sexually active participants are regarded as individuals who reported at least one sexual partner (opposite-sex or same-sex) in the past year. Too few women reported paying for sex to permit a meaningful analysis. PHQ-2=Patient Health Questionnaire-2. STI=sexually transmitted infection.

* Variations from the figure of 20% (cut-off used for low sexual function) indicate increased or decreased sexual function with variable groups.

† A multidimensional measure of area (neighbourhood)-level deprivation based on the participant's postcode; Index of Multiple Deprivation scores for England, Scotland, and Wales were adjusted before assignment to quintiles by use of a method by Payne and Abel;^22^ this approach allowed use of single Index of Multiple Deprivation measure for the three countries.

‡ Two screening questions (scored 0–3 per question; defined here by a total score of 3 or more^24^) assessed depressive symptoms (PHQ-2),^23^ participants were asked whether they had been bothered by feeling down, depressed, or hopeless, and whether they had been often bothered by little interest or pleasure in doing things, in the previous 2 weeks.

§ Menopausal if woman was older than 45 years and had not had a period in more than a year.

¶ Other means easy with a husband or wife or regular partner, but difficult with a new partner; easy with a new partner, but difficult with a husband or wife or regular partner; difficult with any partner, it depends, sometimes easy, and sometimes difficult.

‖ Participants were asked to rate how happy they were in their relationship from 1 (very happy) to 7 (very unhappy); responses of 1 or 2 were regarded as denoting participants who were happy with their relationship.

** First intercourse classified as competent if there was absence of duress and regret about timing; if there was autonomy of decision; and if a reliable form of contraception was used.^25^

†† Defined as vaginal, oral, or anal intercourse.

‡‡ Defined as genital contact not involving vaginal, oral, or anal intercourse, but intended to achieve orgasm, for example stimulating by hand.

§§ Female or male sexual partners, or both.

¶¶ Defined as anyone having sex with you against your will after the age of 13 years.

‖‖ Diagnosed with chlamydia, gonorrhoea, herpes, genital warts, trichomonas, non-specific or non-gonococcal urethritis, or syphilis.

After adjustment for age, low sexual function was associated with unemployment, but not with living in an area of higher deprivation (Table 1, Table 2). We noted strong associations between low sexual function and current depression and with poor self-assessed general health (Table 1, Table 2). Among women, we saw an association with menopausal status and low sexual function, but women who were pregnant in the past year were less likely to have low sexual function than were women who had not been pregnant (table 2).

Compared with individuals who were cohabiting, individuals who had never lived with a partner were more likely to have low sexual function, as were those who had been in a relationship that had ended (Table 1, Table 2). Participants who were not happy with their relationship were more likely to have low sexual function, as were both sexes who did not find it easy to talk about sex with a partner (Table 1, Table 2).

Low sexual function was associated with lack of sexual competence (defined as absence of duress and regret about timing, autonomy of decision, and use of a reliable form of contraception) at first intercourse^25^ and with sexual experience in the past 4 weeks (specifically, having sex fewer than four times, masturbation, and no genital contact without intercourse; Table 1, Table 2). We also noted an association between low sexual function and having a same-sex partner in the past 5 years (Table 1, Table 2). For men, we noted a strong association between low sexual function and paying for sex in the past year (table 1). Among women only, we noted a strong association with reporting higher numbers of lifetime partners (table 2). Low sexual function was also associated with negative sexual health outcomes; most strongly with non-volitional sex but also with diagnosis with a sexually transmitted infection in the past 5 years (Table 1, Table 2).

**Figure 3:**
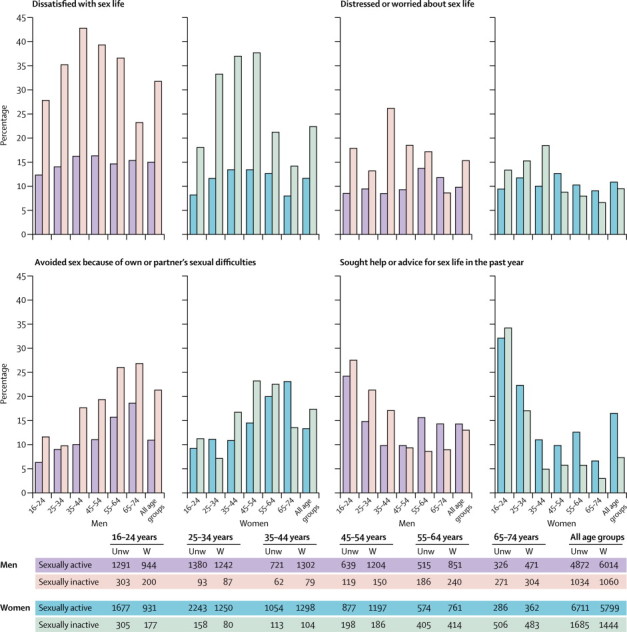
Self-appraisal of sex life by sex, age group, and whether sexually active, in individuals who reported ever having sex Unw=unweighted. W=weighted.

We also report on the proportion of sexually active men and women endorsing items in the first two components of the Natsal-SF, which focus on sexual response problems and the sexual relationship. For component three, appraisal of sex life, we compare reports of sexually active and inactive participants (figure 3).

Table 3 shows the proportion of men and women reporting specific problems with sexual response lasting at least 3 months in the past year. For men, the most commonly reported problems were lack of interest in sex (14·9%), reaching a climax more quickly than desired (14·9%), and difficulty getting or keeping an erection (12·9%). For women, the most common problems were lack of interest in sex (34·2%), difficulty in reaching climax (16·3%), an uncomfortably dry vagina (13·0%), and lack of enjoyment (12·1%). Reporting lack of interest was twice as common among women compared with men. Reporting lack of enjoyment, physical pain and difficulty reaching climax were also more common among women (table 3). In the youngest participants (aged 16–24 years) the most common problem among men was reaching a climax too quickly (16·5%); in women it was lacking interest in sex (24·8%) and difficulty reaching climax (21·0%).

**Table 3:** Percentage of sexually active participants reporting problems with individual sexual response lasting 3 months or more in the past year, by sex and age group

	**16–24 years**	**25–34 years**	**35–44 years**	**45–54 years**	**55–64 years**	**65–74 years**	**All age groups**	p value*
**Men**								
Lacked interest in having sex	11·5% (9·4–14·0)	14·5% (12·7–16·7)	17·2% (14·5–20·4)	15·3% (12·5–18·7)	16·0% (13·0–19·6)	13·6% (10·1–18·0)	14·9% (13·8–16·1)	0·0961
Lacked enjoyment in sex	5·4% (4·2–7·0)	6·7% (5·4–8·3)	5·0% (3·6–7·0)	3·3% (2·1–5·2)	4·6% (3·1–6·9)	1·8% (0·8–3·9)	4·8% (4·1–5·5)	0·0071
Felt anxious during sex	5·7% (4·5–7·1)	6·3% (5·0–7·8)	5·8% (4·2–7·9)	4·4% (3·0–6·4)	5·5% (3·8–8·0)	3·8% (2·3–6·2)	5·4% (4·8–6·2)	0·4269
Felt physical pain as a result of sex	1·8% (1·2–2·9)	1·7% (1·1–2·7)	1·8% (1·1–3·1)	2·0% (1·1–3·5)	1·9% (1·0–3·7)	1·0% (0·2–4·0)	1·8% (1·4–2·3)	0·9243
Felt no excitement or arousal during sex	3·3% (2·4–4·5)	4·3% (3·2–5·9)	3·3% (2·2–4·9)	2·2% (1·3–3·7)	2·6% (1·6–4·3)	2·7% (1·3–5·5)	3·1% (2·6–3·7)	0·2245
Difficultly in reaching climax	9·2% (7·6–11·2)	9·8% (8·1–11·8)	8·3% (6·3–10·8)	7·9% (5·8–10·5)	10·6% (8·2–13·5)	10·4% (7·3–14·5)	9·2% (8·3–10·1)	0·5100
Reached climax more quickly than you would like	16·5% (14·5–18·6)	19·1% (16·7–21·7)	15·8% (13·1–19·0)	13·6% (10·8–17·0)	10·0% (7·5–13·2)	10·8% (7·7–14·8)	14·9% (13·8–16·2)	0·0002
Trouble getting or keeping an erection	7·6% (6·1–9·5)	7·9% (6·4–9·6)	7·9% (5·9–10·4)	13·4% (10·8–16·5)	23·5% (19·8–27·6)	30·0% (25·1–35·4)	12·9% (11·8–14·0)	<0·0001
Experienced one or more of these problems	36·2% (33·3–39·1)	39·7% (36·8–42·6)	40·3% (36·5–44·3)	40·1% (36·1–44·2)	48·1% (43·3–52·9)	53·5% (47·8–59·2)	41·6% (40·0–43·3)	<0·0001
Experienced two or more of these problems	13·6% (11·7–15·7)	14·9% (12·9–17·2)	13·9% (11·3–17·0)	11·7% (9·2–14·7)	15·7% (12·8–19·2)	13·0% (9·7–17·2)	13·8% (12·8–15·0)	0·3685
Denominators†	1291, 944	1380, 1242	721, 1302	639, 1204	515, 851	326, 471	4872, 6014	
**Women**								
Lacked interest in having sex	24·8% (22·6–27·1)	31·9% (29·8–34·1)	37·0% (33·9–40·2)	37·9% (34·6–41·4)	38·8% (34·5–43·2)	34·2% (28·4–40·5)	34·2% (32·8–35·6)	<0·0001
Lacked enjoyment in sex	11·3% (9·7–13·2)	13·2% (11·8–14·8)	11·0% (9·1–13·3)	12·7% (10·5–15·2)	14·2% (11·4–17·7)	8·0% (5·1–12·2)	12·1% (11·2–13·1)	0·0737
Felt anxious during sex	8·2% (6·7–9·9)	8·2% (6·9–9·6)	4·2% (3·1–5·7)	3·6% (2·5–5·1)	2·7% (1·5–4·8)	2·0% (0·8–4·7)	5·2% (4·7–5·9)	<0·0001
Felt physical pain as a result of sex	9·5% (8·1–11·2)	8·0% (6·7–9·4)	5·3% (3·9–7·1)	6·4% (4·9–8·5)	10·4% (7·9–13·4)	5·3% (3·2–8·8)	7·5% (6·7–8·3)	0·0006
Felt no excitement or arousal during sex	8·6% (7·2–10·2)	8·0% (6·9–9·4)	7·1% (5·6–8·9)	8·9% (7·0–11·2)	9·5% (7·3–12·4)	6·9% (4·3–10·9)	8·2% (7·5–9·0)	0·4626
Difficultly in reaching climax	21·0% (18·9–23·4)	17·2% (15·6–19·0)	14·3% (12·2–16·8)	14·7% (12·2–17·5)	16·3% (13·4–19·8)	13·7% (9·9–18·5)	16·3% (15·3–17·3)	0·0029
Reached climax more quickly than you would like	3·8% (2·9–5·0)	2·5% (1·9–3·3)	1·7% (1·0–2·6)	2·6% (1·6–4·0)	1·6% (0·8–3·1)	1·1% (0·4–2·9)	2·3% (1·9–2·8)	0·0136
Uncomfortably dry vagina	9·4% (7·9–11·2)	9·7% (8·4–11·2)	7·5% (6·0–9·5)	14·1% (11·7–16·8)	26·9% (23·2–30·9)	20·0% (15·6–25·3)	13·0% (12·0–14·0)	<0·0001
Experienced one or more of these problems	46·5% (43·8–49·2)	48·5% (46·2–50·8)	49·1% (45·9–52·3)	52·3% (48·8–55·8)	61·5% (57·0–65·7)	55·7% (49·5–61·7)	51·2% (49·8–52·7)	<0·0001
Experienced two or more of these problems	23·0% (20·8–25·4)	23·6% (21·7–25·6)	19·4% (16·9–22·1)	21·9% (19·1–25·0)	27·6% (23·7–31·9)	17·9% (13·6–23·2)	22·4% (21·2–23·6)	0·0028
Denominators†	1677, 931	2243, 1250	1054, 1298	877, 1197	574, 761	286, 362	6711, 5799	

Data are % (95% CI). Sexually active participants are regarded as individuals who reported at least one sexual partner (opposite-sex or same-sex) in the past year.

* χ^2^ p value for association with age-group.

† Unweighted and weighted denominators.

Reporting at least one sexual function problem lasting 3 months or more in the past year was common (41·6% of men and 51·2% of women). Although the proportion of sexually active men and women reporting one or more problem increased steadily with age, this finding was largely due to the age-related increase in erectile difficulties in men (from 7·6% of men aged 16–24 years to 30·0% of men aged 65–74 years) and vaginal dryness in women (from 9·4% to 20·0% across the same age range). We identified two problems that declined with age: reaching a climax too quickly in men (from 16·5% to 10·8%) and anxiety during sex among women (from 8·2% to 2·0%).

**Figure 4:**
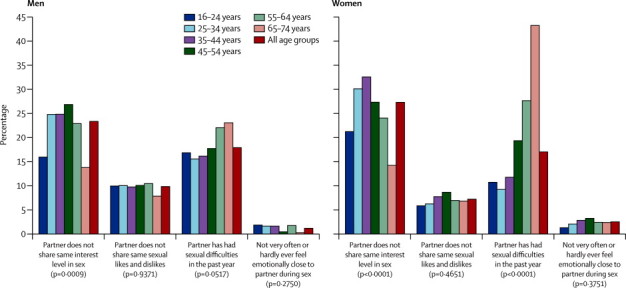
Percentage of participants with particular attitudes towards their sexual partnership, by sex and age group among those who were sexually active and in a sexual relationship lasting the whole year p values correspond to the variation by age group.

Items relating to sexual function within relationships were asked of individuals who were sexually active and in a relationship lasting at least a year before the interview. The most common issue within relationships was an imbalance in level of interest in sex between partners (figure 4; exact figures shown in appendix p 1). We noted small differences between the sexes, with women slightly more likely to report an imbalance in interest (23·4% of men *vs* 27·4% of women, p=0·0010) and not very often or hardly ever feeling emotionally close to their partner (1·3% *vs* 2·6%, p=0·0017), and men being slightly more likely to report not sharing the same sexual likes and dislikes (9·9% *vs* 7·3%, p=0·0006).

Finally, 18·0% of men and 17·1% of women said their partner had had sexual difficulties in the past year. The proportion increased with age, but more so for women than men such that this was reported by almost twice the proportion of women (43·3%) as men (23·1%) in the oldest age group (65–74 years).

Figure 3 shows self-appraisal of sex life in terms of dissatisfaction, distress or worry, avoidance of sex because of own or partner's sexual difficulties, and seeking help or advice among ever-sexually active participants (exact figures shown in appendix p 2).

The proportion of participants expressing dissatisfaction with their sex life was substantially higher in individuals who did not report sex in the past year (sexually inactive) compared with those who did (sexually active): 31·8% for sexually inactive men versus 14·9% for sexually active men and 22·4% for sexually inactive women versus 11·7% for sexually active women. Dissatisfaction varied with age in sexually inactive men and women and sexually active women, but not among sexually active men.

Distress or worry about an individual's sex life was less commonly reported than was dissatisfaction. Distress differed little between sexually active (9·9%) and inactive men (15·4%), and not at all between sexually active (10·9%) and inactive (9·5%) women. For sexually active individuals, the proportion of men reporting distress increased by age, but there was no variation by age for women. In sexually inactive women, the proportion reporting distress declined by age, from 13·4% of those aged 16–24 years to 6·7% of those aged 65–74 years (p=0·0011). In sexually inactive men, the proportion reporting distress declined from 26·2% of those aged 35–44 years to 8·6% of those aged 65–74 years (p=0·0104 for differences across entire age range).

Avoidance of sex because of sexual difficulties was more common in sexually inactive individuals than sexually active individuals (21·4% *vs* 11·0% for men; 17·4% *vs* 13·4% for women). In all groups, avoidance of sex was increasingly common with age, apart from in sexually inactive women, for whom it declined from 23·3% in individuals aged 45–54 years to 13·6% in those aged 65–74 years.

Overall, reported sexual dissatisfaction and avoidance of sex was greatest in individuals who did not report sex in the past year (compared with those who did), but most sexually inactive individuals reported that they were not dissatisfied, distressed, or avoiding sex because of sexual difficulties.

Seeking help or advice for sex lives from any source in the preceding year was more common in sexually active women (16·6%) than in sexually inactive women (7·4%), but this tendency did not differ by sexual activity status in men (14·4% for sexually active *vs* 13·1% for sexually inactive). Irrespective of sexual activity status, help-seeking was more common in younger participants than older ones; with the sources of help in those aged 16–24 years being predominantly informal (data not shown).

## Discussion

To our knowledge, the Natsal-SF is the first measure of sexual function with male and female versions, specifically designed for use in the general population (panel 2). We show wide variability in the distribution of sexual function scores, and we provide the first prevalence estimates of sexual response problems in the British population for 10 years.^5^

Panel 2 **Research in context**

**Systematic review**

We searched Pubmed, BIDS, Psychinfo, Medline, and the Cochrane Database for articles published in English, with a wide range of search terms including “sexual function/dysfunction”, “sexual satisfaction/dissatisfaction”, “sexual difficulties”, “psychosexual disorder*/problem*”, “sexual relationship”, “sexual distress”, “classif*”, “measure*”, “model”, “prevalence”, “incidence”, and “epidemiol*”.^26^ We noted a focus on clinically defined problems with sexual response, and a tendency to separate measurement of problems from other aspects of function relevant to everyday life, such as the sexual relationship, the level of satisfaction, and the significance of problems for the individual concerned. We searched for, but did not find, a brief measure of sexual function with male and female versions, suitable for use in general population samples and including items on the sexual relationship, satisfaction and distress. Thus we developed and validated a new measure—the Natsal-SF—specifically for the third National Survey of Sexual Attitudes and Lifestyles (Natsal-3). Despite the fact that sexual function is fundamental to sexual health,^27^ we also did not find any studies exploring the link between low sexual function and other aspects of sexual health. As a population-based survey of sexual health broadly defined, Natsal-3 was well placed to explore these associations.

**Interpretation**

We aimed to explore the distribution of sexual function using a definition of function that is relevant to everyday life. We showed a wide variability in sexual function across the life course. We noted no empirical threshold (cutoff) to denote low sexual function in the lower end of the distribution of scores on the Natsal-SF. This finding confirms our strategy of treatment of sexual function as a continuum of experience. We reported that low sexual function was associated with other indicators of poor sexual health, and call for greater attention to be paid to low sexual function within broader sexual health policies, interventions, and services.

We show that sexual response problems lasting at least 3 months in the preceding year are common, even in young people. More than 40% of men and 50% of women report one or more problems, but the proportion of sexually active individuals reporting distress about their sex life is much lower (about 10%). Our estimates of individual problems include infrequent and frequent symptoms as well as mild and bothersome problems and should be interpreted accordingly.

Low sexual function is associated with increasing age but the strength of the association does not increase beyond the 55–64-year-old age group. Our data suggest variation in sexual function with aspects of life stage and life events (eg, employment status, pregnancy, and relationship status). Our data also show associations between low sexual function and other sexual health outcomes such as diagnosis of sexually transmitted infection^28^ and non-volitional sex.^29^ We also show strong associations between low sexual function and many of the factors associated with these outcomes, such as higher number of sexual partners over the lifetime, paying for sex, and reporting same-sex partners. The reasons for the association with reporting same-sex partners are as yet inadequately understood.^30^

Limitations of the study related to response rate are addressed elsewhere.^18^, ^19^ Natsal-3 relies on self-reported data, which are subject to recall and desirability bias. Questions about sexual function are sensitive and problems might be prone to under-reporting. We sought to minimise this bias by use of computer-assisted self-interview technology^31^ and by describing sexual function problems as common difficulties. Because these data are cross-sectional, we cannot infer causality in the associations we show, and in the case of many of the factors linked with low sexual function, two-way causality is a distinct possibility.

The distribution of scores on Natsal-SF had no empirical threshold to define low sexual function. Consistent with approaches used in many composite health and socioeconomic measures,^22^ we used the lowest quintile of the distribution of the Natsal-SF to denote low sexual function. We did a sensitivity analysis and confirmed that the associations we noted for Natsal-SF hold true using cutoffs varying between 5% and 35% (data not shown).

With the exception of items relating to appraisal of sex life, our analysis was restricted to individuals who were sexually active in the past year, and so excluded individuals who may not have had sex recently because of sexual difficulties. The levelling-off of the age-related decline in sexual function after 55 years could reflect the fact that men and women with better sexual function are more likely to remain sexually active, giving rise to survivor bias and a possible underestimate of sexual response problems. However, if this explanation were the whole reason, we could expect distress and dissatisfaction to be higher in older people who are not sexually active, but we did not find this association. A further explanation might be that people adjust their priorities and practices to cope with changes in physiological response, health, and partnership problems;^32^ and they could revise their expectations downwards in later years.

In Natsal-2, we reported on a smaller range of sexual function problems than in Natsal-3 using the Natsal-SF, and this study builds on this previous work.^5^, ^33^ Estimates between the two surveys are not strictly comparable because of differences in item wording and filtering. In general, differences in sampling and definition of problems make comparison between prevalence studies very difficult.^34^, ^35^ With this caveat, a recent review of epidemiological studies,^35^ established across studies, a range of prevalence estimates for individual problems; our estimates all fall within these ranges. Of note, our estimate of lack of interest in men falls at the higher end of the range (14·9% of men in our study; range across studies 8–18%), as does our estimate of difficulty reaching climax (9·2% of men in our study; range 1–10%).^35^

Our findings will be of interest to public health practitioners and researchers. In terms of research, the sex-specific cutoffs we imposed to show low function (the lowest quintile) provide comparators that can be used in future studies, although our particular thresholds are specific to the general British population aged 16–74 years.

Our data have implications for the targeting of interventions to address problems with sexual function. The sizeable prevalence of sexual problems in young people calls for provision of appropriate advice and help aimed at improving the quality of their sexual experience.^36^, ^37^ A greater emphasis is needed on sexual wellbeing in educational interventions and provision of services designed for young people.^37^ Because lack of sexual competence at first intercourse is a risk factor for low subsequent sexual function, interventions need to be in place before onset of sexual activity.

Our data also stress the importance of considering sexual function within the broader context of the sexual relationship.^1^, ^4^ We noted that low sexual function was associated with relationship breakdown, relationship unhappiness, and with difficulty talking about sex. One in four men and women report not sharing the same level of interest in sex as their partner. One in five participants of our survey has a partner with sexual difficulties, and the proportions increase with age particularly among women. Nearly half the women in the oldest age group are affected, in part because male partners are increasingly susceptible to erectile difficulties as they age.^38^ Cross-sectional data cannot shed light on the extent to which low sexual function contributes to relationship problems, and the extent to which the reverse is true, but each has clinical and public health significance and more research is needed on the pathways and mechanisms at work.

Several associations noted in our study will be of interest to practitioners exploring the causes and treatment of sexual function problems in patients. The association between low sexual function and unemployment has been noted in other studies, which have suggested that problems of self-esteem and depression among those out of work might be contributory factors.^39^, ^40^ The strong link we show between sexual function, depression, and self-reported health status is also established in the literature^1^, ^41^, ^42^ and suggests that routine enquiry about sexual problems among those with mental and general health conditions could be warranted. In terms of treatment, genital contact without intercourse is associated with better sexual function, suggesting that a focus on physical intimacy might be helpful, especially in couples with problems that preclude penetrative sex.^43^ Although the experience of sexually inactive individuals is less well documented, findings from studies among women in the community^44^, ^45^ concur with our finding that large proportions of sexually inactive individuals are not dissatisfied, distressed, or avoiding sex because of sexual difficulties. These data caution against assumptions that sexual inactivity is in itself problematic and need to be taken into account in the provision of services and treatment options.

The links between reduced sexual function and other sexual health outcomes make a strong case for greater focus on sexual function within the context of sexual health. In research, sexual function could be included in quality of life measures and as an endpoint in studies assessing the success of sexual health interventions; in education, the sexual health curriculum could include positive as well as negative aspects of sexual experience; and in clinical practice, increased efforts could be made to address sexual problems within services for diagnosis and care of sexually transmitted infections,^46^ as well as in primary and secondary care.

The Natsal-SF is novel in providing a composite assessment of sexual function. By measuring the extent to which an individual is able to participate in and enjoy a sexual relationship across a large representative sample, we showed how sexual function varies across the population and through the life course. Our hope is that the Natsal-SF will encourage a move away from measurement approaches that overmedicalise sexual function, towards those that take account of the variability of sexual function experience in the population, and the personal significance of sexual function problems for men, women, and their partners.

## Supplementary Material

Supplementary appendix

Supplementary video Results of over 45,000 respondents, recording the dramatic changes in our sexual lifestyles over the past 60 years.
