# Supplementary material for: Sexual function in Britain: findings from the third National Survey of Sexual Attitudes and Lifestyles (Natsal-3)
Source: Lancet. 2013 Nov 30;382(9907):1817–29. doi: 10.1016/S0140-6736(13)62366-1 (PMC3898902; doi:10.1016/S0140-6736(13)62366-1)
Supplement: Supplementary appendix [file mmc1.pdf]

THE LANCET

Supplementary appendix

This appendix formed part of the original submission and has been peer reviewed. We post it as supplied by the authors.

Supplement to: Mitchell KR, Mercer CH, Ploubidis GB, et al. Sexual function in Britain: findings from the third National Survey of Sexual Attitudes and Lifestyles (Natsal-3). *Lancet* 2013; published online Nov 26. http://dx.doi.org/10.1016/S0140-6736(13)62366-1.

Web appendix 1: Percentage of participants with particular attitudes towards their sexual partnership, by sex and age group among those who were sexually active[1] and in a sexual relationship lasting the whole year (data[2] for figure 4)

	16-24	25-34	35-44	45-54	55-64	65-74	All	p-value[3]
**MEN**								
**Partner does not share same interest level in sex**	16.0% (12.2-20.8)	24.9% (22.1-28.0)	24.9% (21.3-29.0)	26.9% (22.6-31.7)	23.0% (18.8-27.9)	13.8% (9.9-18.9)	23.4% (21.7-25.2)	0.0009
**Partner does not share same sexual likes and dislikes**	10.0% (7.3-13.5)	10.1% (8.1-12.5)	9.8% (7.4-12.9)	10.1% (7.6-13.4)	10.5% (7.6-14.1)	7.9% (5.0-12.3)	9.9% (8.7-11.2)	0.9371
**Partner has had sexual difficulties past year**	16.9% (13.2-21.3)	15.6% (13.2-18.3)	16.2% (13.0-20.0)	17.8% (14.2-22.1)	22.1% (17.9-27.1)	23.1% (17.8-29.5)	18.0% (16.4-19.7)	0.0517
**Hardly ever or not very often feel emotionally close to partner during sex**	1.9% (1.0-3.6)	1.7% (0.9-3.1)	1.7% (0.8-3.3)	0.5% (0.2-1.6)	1.8% (0.6-5.0)	0.3% (0.0-2.2)	1.3% (0.9-1.9)	0.2750
**Denominators[4]**	428, 332	908, 867	538, 1053	466, 951	384, 679	237, 356	2961, 4239	
**WOMEN**								
**Partner does not share same interest level in sex**	21.3% (18.2-24.7)	30.1% (27.5-32.7)	32.6% (29.4-36.0)	27.4% (24.0-31.1)	24.1% (20.1-28.7)	14.3% (10.1-19.9)	27.4% (25.9-28.9)	<0.0001
**Partner does not share same sexual likes and dislikes**	5.9% (4.3-8.1)	6.3% (5.1-7.9)	7.8% (6.1-10.0)	8.7% (6.6-11.4)	7.0% (4.9-9.9)	6.9% (3.9-11.9)	7.3% (6.5-8.3)	0.4651
**Partner has had sexual difficulties past year**	10.8% (8.5-13.6)	9.3% (7.8-11.0)	11.8% (9.5-14.4)	19.4% (16.5-22.6)	27.7% (23.3-32.6)	43.3% (36.5-50.4)	17.1% (15.8-18.4)	<0.0001
**Hardly ever or not very often feel emotionally close to partner during sex**	1.4% (0.8-2.5)	2.1% (1.4-3.0)	2.9% (1.9-4.4)	3.3% (2.2-5.0)	2.5% (1.4-4.7)	2.5% (1.0-6.0)	2.6% (2.1-3.2)	0.3751
**Denominators[4]**	958, 527	1546, 912	803, 1060	665, 979	419, 590	204, 268	4595, 4337	

[1] Sexually active participants are regarded as individuals who reported at least one sexual partner (opposite-sex or same-sex) in the past year.
[2] Data are % (95% CI)
[3] χ^2 p value for association with age-group.
[4] Unweighted and weighted denominators.

Web appendix 2: Self-appraisal of sex life by sex, age group, and whether sexually active[1], in individuals who reported ever having sex (data[2] for figure 3)

	16-24	25-34	35-44	45-54	55-64	65-74	All	p-value[3]
**MEN**								
**Dissatisfied with sex life**								
Sexually Active	12.3%	14.0%	16.2%	16.3%	14.7%	15.4%	14.9%	0.3256
	(10.4-14.6)	(12.2-16.0)	(13.6-19.2)	(13.4-19.7)	(11.8-18.3)	(11.7-20.0)	(13.8-16.1)	
Sexually Inactive	27.8%	35.2%	42.8%	39.3%	36.6%	23.2%	31.8%	0.0034
	(22.3-34.0)	(25.9-45.9)	(29.7-56.9)	(30.4-49.1)	(29.5-44.4)	(18.1-29.1)	(28.7-35.1)	
**Distressed or worried about sex life**								
Sexually Active	8.5%	9.5%	8.5%	9.3%	13.8%	11.8%	9.9%	0.0159
	(7.0-10.3)	(8.0-11.2)	(6.6-10.8)	(7.1-12.0)	(11.0-17.2)	(8.6-16.0)	(9.0-10.8)	
Sexually Inactive	17.9%	13.2%	26.2%	18.5%	17.2%	8.6%	15.4%	0.0104
	(13.4-23.6)	(7.5-22.3)	(15.8-40.1)	(11.9-27.7)	(11.8-24.5)	(5.5-13.0)	(13.1-18.1)	
**Avoided sex because of sexual difficulties**								
Sexually Active	6.4%	9.1%	10.1%	11.1%	15.8%	18.7%	11.0%	<0.0001
	(5.1-8.1)	(7.5-10.9)	(8.0-12.7)	(8.7-14.2)	(12.7-19.5)	(14.6-23.6)	(10.0-12.1)	
Sexually Inactive	11.7%	9.8%	17.7%	19.4%	26.1%	26.9%	21.4%	0.0084
	(7.0-19.0)	(5.1-17.9)	(9.4-31.0)	(11.4-30.9)	(19.7-33.8)	(21.6-33.1)	(18.4-24.6)	
**Sought help or advice for sex life**								
Sexually Active	24.3%	14.9%	9.9%	9.9%	15.7%	14.4%	14.4%	<0.0001
	(21.9-26.9)	(13.0-17.1)	(7.8-12.4)	(7.7-12.6)	(12.7-19.3)	(11.0-18.5)	(13.3-15.4)	
Sexually Inactive	27.6%	21.4%	17.2%	9.4%	8.7%	9.0%	13.1%	0.0002
	(20.4-36.3)	(14.2-31.0)	(7.9-33.5)	(4.3-19.4)	(5.0-14.4)	(5.9-13.6)	(10.8-15.9)	
**Sexually Active denominators[4]**	1291, 944	1380, 1242	721, 1302	639, 1204	515, 851	326, 471	4872, 6014	
**Sexually Inactive denominators[4]**	303, 200	93, 87	62, 79	119, 150	186, 240	271, 304	1034, 1060	
**WOMEN**								
**Dissatisfied with sex life**								
Sexually Active	8.2%	11.6%	13.4%	13.4%	12.7%	8.0%	11.7%	0.0035
	(6.9-9.8)	(10.2-13.1)	(11.2-15.8)	(11.2-15.9)	(10.0-15.9)	(5.0-12.5)	(10.8-12.7)	
Sexually Inactive	18.1%	33.3%	37.0%	37.7%	21.2%	14.2%	22.4%	<0.0001
	(13.9-23.3)	(26.0-41.6)	(27.2-47.8)	(30.0-46.1)	(17.0-26.2)	(11.2-17.8)	(20.3-24.8)	
**Distressed or worried about sex life**								
Sexually Active	9.5%	11.8%	10.1%	12.7%	10.3%	9.1%	10.9%	0.1832
	(7.9-11.3)	(10.4-13.4)	(8.4-12.1)	(10.4-15.3)	(7.9-13.2)	(6.1-13.3)	(10.0-11.8)	
Sexually Inactive	13.4%	15.3%	18.5%	8.8%	8.0%	6.7%	9.5%	0.0011
	(9.5-18.6)	(10.0-22.8)	(11.5-28.4)	(5.3-14.2)	(5.5-11.5)	(4.6-9.6)	(8.1-11.2)	
**Avoided sex because of sexual difficulties**								
Sexually Active	9.3%	11.2%	10.9%	14.6%	20.0%	23.2%	13.4%	<0.0001
	(7.7-11.2)	(9.8-12.8)	(9.1-13.1)	(12.2-17.3)	(16.7-23.9)	(18.3-28.9)	(12.5-14.5)	
Sexually Inactive	11.3%	7.2%	16.8%	23.3%	22.6%	13.6%	17.4%	0.0008
	(6.2-19.7)	(4.0-12.6)	(9.7-27.4)	(17.2-30.9)	(18.0-28.0)	(10.5-17.4)	(15.2-19.8)	
**Sought help or advice for sex life**								
Sexually Active	32.2%	22.4%	11.1%	9.9%	12.7%	6.7%	16.6%	<0.0001
	(29.7-34.8)	(20.5-24.4)	(9.3-13.3)	(7.9-12.3)	(10.1-15.9)	(4.4-10.3)	(15.6-17.6)	
Sexually Inactive	34.3%	17.1%	5.0%	5.8%	5.8%	3.1%	7.4%	<0.0001
	(26.0-43.7)	(11.3-25.1)	(2.0-11.9)	(2.8-11.6)	(3.6-9.3)	(1.7-5.5)	(6.0-9.1)	
**Sexually Active denominators[4]**	1677, 931	2243, 1250	1054, 1298	877, 1197	574, 761	286, 362	6711, 5799	
**Sexually Inactive denominators[4]**	305, 177	158, 80	113, 104	198, 186	405, 414	506, 483	1685, 1444	

[1] Sexually active participants are regarded as individuals who reported at least one sexual partner (opposite-sex or same-sex) in the past year.
[2] Data are % (95% CI)
[3] χ^2 p value for association with age-group.
[4] Unweighted and weighted denominators.
